# Giving Rest to the Restless Leg: A Case Report of How Self-Education Prepending Web-Based Interventions Can Ameliorate Restless Leg Syndrome

**DOI:** 10.7759/cureus.32805

**Published:** 2022-12-21

**Authors:** Qaisar Ali Khan, Hoor ul Ain, Kavya Shah, Karma Patel, Christopher Farkouh, Amba Swamy Naidu Pasupuleti, Bhavana Vattikuti, Vinay Suresh, Natalia Santiago, Matthew Farkouh

**Affiliations:** 1 Internal Medicine, DHQ Teaching Hospital Kohat, Kohat, PAK; 2 Internal Medicine, Jinnah Medical College, Peshawar, PAK; 3 Medicine, Gujarat Medical Education and Research Society Medical College and Hospital, Himmat Nagar, IND; 4 Medicine, Motilal Nehru Medical College and SRN Hospital, Prayagraj, IND; 5 General Medicine, Rush Medical College, Chicago, USA; 6 Medicine, MNR Medical College and Hospital, Sangareddy, IND; 7 Internal Medicine, Cebu Doctors University College of Medicine, Philippines, PHL; 8 General Medicine, King George’s Medical University, Lucknow, IND; 9 General Medicine, Universidad Autonoma de Guadalajara, Guadalajara, MEX; 10 General Medicine, Ponce Health Sciences University, Ponce, USA

**Keywords:** self- education, amelioration, case report, web- based intervention, restless leg syndrome

## Abstract

Restless leg syndrome (RLS) is a neurological disorder characterized by an irresistible urge to move one's leg sporadically. The pathogenesis of RLS, also known as Willis Ekborn disease, is not fully understood; however, scientists note a complex interplay between multiple neuronal pathway-related genes with endogenous and exogenous factors. We report a case of a previously healthy 27-year-old man complaining of a continuous urge to move his right leg, notably at night. Laboratory evaluation proved negative for secondary causes of RLS; hence the condition was labeled as “primary idiopathic.” The patient was started on appropriate pharmacotherapy and was advised to self-educate regarding his ailment. The patient began internet-based self-education and displayed excellent improvements on the International Restless Leg Syndrome Scale (IRLS). Mental exercises, such as self-education using web-based intervention and pharmacotherapy, could alleviate factors in patients with primary idiopathic RLS. Further research is needed to clarify self-education's role in managing RLS.

## Introduction

Restless leg syndrome (RLS), also known as Willis Ekborn disease, is a movement disorder characterized by an uncontrollable urge to move the legs, leading to impaired sleep patterns and quality of life [1]. RLS affects nearly 15% of the global population, and symptoms commonly present in the evening or at night when a person is resting [2]. The pathogenesis of RLS is idiopathic and can also be associated with conditions such as iron deficiency anemia, pregnancy, chronic kidney disease, and active smoking [3]. Infections such as COVID-19 have also been noted to cause sleep disturbances and RLS through complex interactions between the human immune response and the central nervous system [2,3]. Non-pharmacological measures can be utilized in conjunction with pharmacological measures to lead to better outcomes [4]. Non-pharmacological measures include walking, massaging the area, stretching, cognitive distractions (such as knitting or completing puzzles), or taking a contrast bath [4]. Patients should be educated regarding the importance of proper sleep hygiene and regular sleep-wake cycles consisting of an adequate duration of sleep. Exercise, yoga, and lavender-oil massages may also be helpful [4]. Pharmacological measures that have proven beneficial include iron supplementation, dopamine agonists like pramipexole, ropinirole, gabapentin, and opioid agonists like oxycodone, tramadol, and methadone [5]. The psychological effects of presenting educational videos regarding RLS to patients are significant in reducing the severity of symptoms and leading to improved sleep, mood, quality of life, heart rate, and blood pressure [6]. This beneficial effect is also evident when patients engage in mind-dispersion-based activities such as yoga, especially in aged women [6,7].

## Case presentation

A 27-year-old male patient presented to the physician's clinic complaining of a continuous urge to move his right leg for two weeks. He noticed more prominent symptoms at night, leading to continual distress and poor sleep. The patient does not smoke or drink alcohol. He did not have tendencies for depression or anxiety. Based on the medical history, complete physical and neurological examination, and blood tests the patient was diagnosed with a case of primary idiopathic RLS one year back (Table 1).

**Table 1:** Laboratory evaluation Hb: Hemoglobin; MCV: mean corpuscular volume; RDW: red cell distribution width; g/dL: grams per deciliter; fL: femtoliter; μg/L: microgram per liter; mg/dL: milligrams per deciliter; ng/mL: nanograms per milliliter.

investigation	Result	Normal range
Hb	13.4	13-17 g/dL
MCV	94.1	80-100 fL
RDW	12.1	11.8-14.5%
serum ferritin	185	24-285 μg/L
Serum creatinine	0.9	0.74 to 1.35 mg/dL
Cholecalciferol	32.4	20-40 ng/mL
Serum calcium	8.7	8.6-10.3 mg/dL
Albumin	4.8	3.4 to 5.4 g/dL

**Figure 1:**
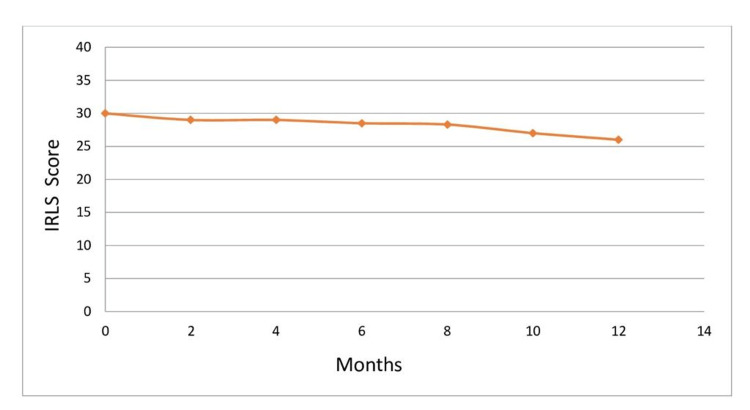
The responses of the patient to pharmacotherapy-only intervention on the IRLS scale for 12 months IRLS: International Restless Leg Syndrome Scale

The patient was taking a combination of carbidopa/levodopa 25/250 mg (one tablet once daily) followed by regular exercise recommended by their physician, yet his symptoms did not improve. The patient is a nonsmoker and does not drink alcohol. His past medical history is significant for tuberculosis for which he completed a course of anti-tuberculosis therapy five years ago with complete recovery. Family history was significant for mental retardation in the youngest brother. The patient's score on the International Restless Leg Syndrome (IRLS) scale was decreased by 4 points (from 30 to 26) from the previous year (Figure 1).

**Figure 2:**
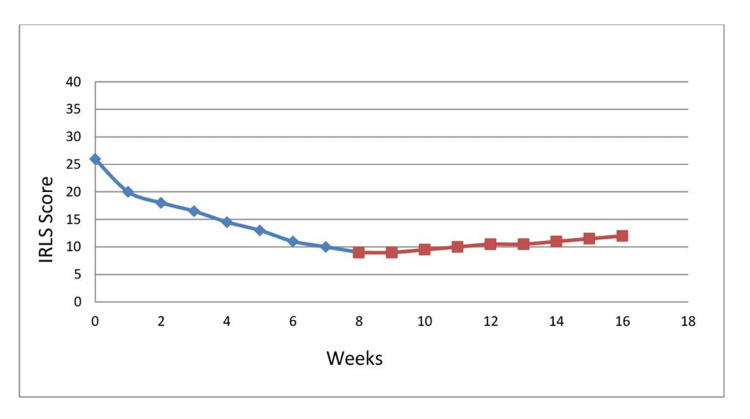
The responses of the patient on the IRLS scale to pharmacotherapy in addition to self-education at 8- and 16-week follow-up visits IRLS: International Restless Leg Syndrome

The patient appeared frustrated and frequently moved his legs sporadically during the examination. The patient was advised to continue taking carbidopa/levodopa combination 25/250 mg once daily and, in addition, he was advised to self-educate himself through various sources such as open-access medical journals and the internet. the patient started learning about the pathogenesis, risk factors, treatment and prognosis of RLS using the web. The patient attended two follow-up visits at eight and 16 weeks after starting both interventions. The patient scored nine out of 40 at the eight-week follow-up and 12 out of 40 at the 16-week follow-up on the IRLS scale. The patient showed 52.5% improvement from the baseline in the first eight weeks and then only 7.5% improvement in the next eight weeks because he stopped self-education during this period (Figure 2). 

## Discussion

RLS follows a typical circadian rhythm that is worsened during the night and improved during the daytime. RLS can be primarily idiopathic or secondary, with the causes of secondary RLS entailing three main components: genetic, micronutrient deficiencies such as iron, and neurotransmitter (dopamine) deficiencies in the brain [8]. Patients who have high-stress levels or high physical activity before sleeping tend to have poor sleep quality, augmenting the symptoms of RLS. Using stimulants like caffeine in excessive amounts can add to the degree of anxiety and insomnia patients of RLS are already afflicted with. RLS patients are more likely to be associated with depressive disorders [9]. Poor quality sleep due to RLS can be linked to bouts of depression [9]. Intellectualization, a common psychological defense mechanism, describes reasoning used to resolve a conflict or mitigate anxiety through the acquisition of knowledge in an attempt to give rational form or context to a situation [10]. By applying intellectualization, factors that exacerbate anxiety, such as stress, fear of the unknown, and overthinking, could be reduced in RLS patients.

The IRLS scale is used for the classification of RLS into categories such as “mild,” “moderate,” “severe,” and “very severe” [11]. The RLS rating scale examination asks the patient to rate their symptoms to a standard set of 10 questions. Every question has five response choices scored from “no RLS/impact” (scored as 0) to “very severe RLS/impact” (scored as 4). The intensity of RLS is determined based on the score obtained by the patient with “very severe RLS” ranging from 31 to 40 points, “severe” from 21 to 30 points, “moderate” from 11 to 20 points, and “mild” from 1 to 10 points [11]. Eight weeks after combined pharmacological (carbidopa-levodopa) and web-based interventions, the patient's score reduced from 26 (“severe”) to 9 points (“mild”). However, in our case report, the patient's lab results, and examination were not significant for any of the etiologies; hence, the etiology of his RLS was determined to be idiopathically associated with psychological problems.

Our patient represents a unique case of RLS as he responded well within eight weeks to both interventions. Figure 2 denotes the patient’s symptoms recurred after eight weeks once the patient stopped self-education practices. It is evident from Figure 2 that long-term self-education is necessary to yield a satisfactory response. Therefore, new methods of self-education or intellectualization should be advised. Unfortunately, the internet is not easily available everywhere and the limitation of such interventions is it is not suitable for patients with low income and decreased education [10].

Web-based learning provides an effective way for individual learning, but it may fail to respond well to the individual needs of the learner. There are certain limitations with web-based studies, namely accessibility, and usage, as individuals with low literacy levels could struggle to understand the content available for self-education. Although web-based interventions can be an effective approach in educating literate and competent individuals regarding RLS.

## Conclusions

Self-education can be used as an adjunct to conventional pharmacotherapy in RLS for better outcomes. Web-based intervention, as per the patient's educational level and competency can prove to be an effective method in self-educating patients about their RLS in an understandable manner. Further research is needed to explore other methods by which patients with low levels of literacy or competency can get self-education regarding RLS.
